# Global landscape analysis of clinical trials on gut microbiota modulation therapies for irritable bowel syndrome

**DOI:** 10.3389/fmed.2026.1737537

**Published:** 2026-03-02

**Authors:** Yiting Luo, Junjie Cao, Bingbing Li, Junwen Wang, Tianxiang Geng, Zining Luo, Jiebin Xie

**Affiliations:** 1 Affiliated Hospital of North Sichuan Medical College, Nanchong, Sichuan, China; 2 North Sichuan Medical College, Nanchong, Sichuan, China; 3 Faculty of Dentistry, The University of Hong Kong, Hong Kong SAR, China; 4 Department of Orthopedics, Yantai Yuhuangding Hospital Affiliated to Medical College of Qingdao University, Yantai, Shandong, China

**Keywords:** clinical trial landscape, fecal microbiota transplantation, gut microbiota, irritable bowel syndrome, probiotics

## Abstract

**Objective:**

Systematically analyze the global landscape of interventional clinical trials on gut microbiota modulation (GMM) therapies for irritable bowel syndrome (IBS).

**Methods:**

Searched the Trialtrove database (1998–July 2025) with the key term combination “(Disease is Autoimmune/Inflammation: Irritable Bowel Syndrome) AND (Mechanism of Action: Microbiome modulator)”, included 305 interventional trials (excluded 15 observational studies). Descriptive analysis was done via SPSS 26.0, adhering to TITAN Guidelines 2025.

**Results:**

Asia was the most active region; trials peaked in 2021, with Phase II (44.3%) and IV (33.3%) dominant. Probiotics led (single-strain: Lactobacillus/Bifidobacterium; multi-strain: Lactobacillus + Bifidobacterium), followed by fecal microbiota transplantation (FMT). IBS-D (49.6%) was the main subtype (IBS-C: 26.1%); probiotics were the most frequently studied for both, FMT for IBS-D, and prebiotics for IBS-C.

**Conclusion:**

GMM therapies for IBS are relatively mature. Personalized therapies are necessary; multiomics and emerging therapies (e.g., *Akkermansia muciniphila*) will promote IBS precision medicine.

## Introduction

Irritable bowel syndrome (IBS) is a prevalent functional gastrointestinal disorder affecting 5–10% of the global population (1). According to the Rome IV criteria, IBS is classified into four subtypes: diarrhea-predominant [IBS-D, the most prevalent (2)], constipation-predominant (IBS-C), mixed (IBS-M), and unclassified (IBS-U). This disorder is characterized by recurrent abdominal pain and altered bowel habits (1), significantly impairing patients’ quality of life.

In recent years, gut microbiota modulation (GMM) therapies—including probiotics, fecal microbiota transplantation (FMT), prebiotics, postbiotics, and synbiotics—have emerged as promising therapeutic strategies for IBS. These interventions exert their effects through mechanisms such as restoring gut microbial homeostasis and repairing intestinal mucosal barrier integrity (3), showing potential in alleviating core IBS symptoms (e.g., abdominal pain, bloating, and bowel movement abnormalities). For example, *Lactobacillus plantarum* 299v reduces abdominal pain and bloating (4).

## Methods

We systematically searched the Trialtrove database, an authoritative global clinical trial registry that integrates data from dozens of primary registries, including ClinicalTrials.gov and the WHO International Clinical Trials Registry Platform (ICTRP). The search was conducted on July 9, 2025, covering records from 1998 onward. We utilized the database’s built-in classification filters, with the specific search syntax being: (Disease is Autoimmune/Inflammation: Irritable Bowel Syndrome) AND (Mechanism of Action: Microbiome modulator). No additional filters (e.g., by trial phase, intervention type, or recruitment status) were applied to ensure a broad capture of all relevant trials. The search was not restricted by language or geographical region.

Two investigators independently screened all retrieved records. The inclusion scope of this analysis was defined as all interventional trials evaluating GMM therapies for IBS. Accordingly, from the initial set of 320 identified records, 15 observational studies were excluded. During the screening process, no records were identified for exclusion due to duplicate registration or insufficient core information. A total of 305 interventional trials were ultimately included in the final analysis. Metrics analyzed included trial status, geographic distribution, temporal trends, therapy classifications, disease subtypes, and associated therapy selections.

All classifications were primarily based on the original database records and were standardized with reference to international consensus frameworks such as the World Gastroenterology Organisation (WGO) Global Guidelines. A mutually exclusive primary categorization was employed. Each trial was assigned to a single dominant therapy category (e.g., probiotic, fecal microbiota transplantation, synbiotic) for the main analysis. For multi-component interventions: Combinations meeting standard definitions (e.g., probiotic + prebiotic) were classified as “Synbiotics.” Non-standard combinations (e.g., probiotic + herbal medicine) were assigned to created categories (e.g., “Probiotic Combination Therapy”). Regarding IBS subtypes: Subtype classification strictly adhered to the trial registry entries. Trials explicitly designated as diarrhea-predominant (IBS-D), constipation-predominant (IBS-C), mixed (IBS-M), or unsubtyped (IBS-U) were categorized accordingly. Trials registered only for “IBS” without further specification were labeled as “Unspecified.” These were included in summaries of overall trends but were excluded from direct comparative analyses between specific subtypes. Any discrepancies between investigators were resolved through discussion and third-party adjudication. For statistical analysis, descriptive statistics were primarily used, and all statistical analyses were performed using SPSS 26.0. Frequencies of categorical variables (e.g., trial status, therapy classifications, disease subtypes) were calculated and presented as numbers and percentages (*n*, %). Graphical representations were generated to visualize the distribution and trends of the data. All procedures adhered to the TITAN Guidelines 2025.

## Results

**Figure 1:**
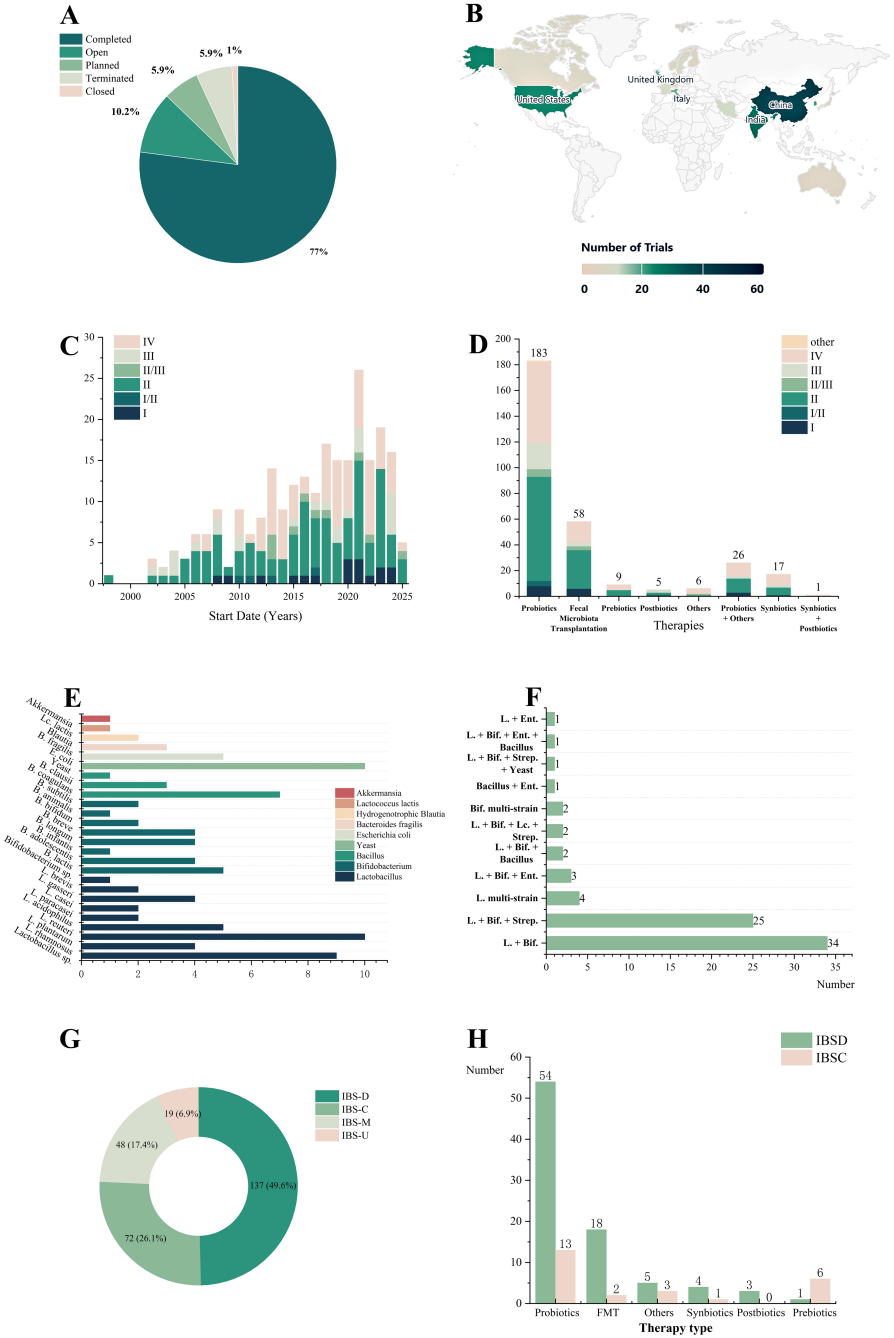
Analysis and trends in clinical trials of gut microbiota modulation therapies for irritable bowel syndrome. **(A)** Distribution of clinical trial status. **(B)** Geographic distribution of clinical trials (only top 20 countries by trial count are shown). **(C)** Annual distribution of IBS clinical trials by phase, from 1998 to July 9, 2025 (59 trials lacking temporal data excluded). **(D)** Distribution of various therapy types used in clinical trials. **(E)** Application of single-strain probiotics. **(F)** Application of multi strain probiotic combinations. **(G)** Distribution of patient subtypes. **(H)** Comparative distribution of therapy selection between IBS-D and IBS-C. *Lc*., *Lactococcus*; *B. fragilis*, *Bacteroides fragilis*; *E. coli*, *Escherichia coli*; *B. clausii*, *Bacillus clausii*, *B. coagulans*, *Bacillus coagulans*; *B*., *Bacillus*; *L*., *Lactobacillus*, *Ent*., *Enterococcus*; *Bif*., *Bifidobacterium*; *Strep*., *Streptococcus*; Multistrain, multistrain combination.

Our landscape analysis of 305 interventional trials yielded several key findings. The results indicate that completed trials predominated (77.0%, Figure 1A). The complete list of all included trials is provided in Supplementary Table 1. Geographically, Asia (China, India, South Korea, etc.) was the most active region, contributing 119 trials (39.0%), followed by North America (32 trials, 10.5%) and Europe (Figure 1B).

In terms of temporal trends, the annual distribution of trials showed an overall increasing trend, peaking in 2021 (Figure 1C). Analysis of trial phases revealed that Phase II (44.3%) and Phase IV (33.3%) trials constituted the majority.

Further analysis of therapy types (Figure 1D) indicated that probiotics were the dominant category, comprising 60.0% of all trials. The majority of probiotic trials were in Phase II (44.3%), and probiotics remained the most common intervention in Phase IV trials (constituting 59.4% of Phase IV trials). Fecal microbiota transplantation (FMT) represented the second-largest category (19.0%), with its trials also concentrated in Phase II (51.7%), followed by Phase IV (27.6%). Notably, combination therapies (e.g., probiotics with herbal medicine, antispasmodics, or prebiotics) are gaining attention.

Regarding specific probiotic strategies, single-strain applications (Figure 1E) most commonly involve *Lactobacillus* (e.g., *Lactobacillus plantarum*), followed by *Bifidobacterium*, *Bacillus* and *Saccharomyces*. Among the multistrain formulations (Figure 1F), *Lactobacillus* + *Bifidobacterium* combinations were the most prevalent, followed by *Lactobacillus* + *Bifidobacterium* + *Streptococcus* (e.g., the VSL#3 formulation^1^).

To advance personalized therapy, we analyzed patient subtypes (Figure 1G). IBS-D (49.6%) was the primary focus, nearly double that of IBS-C (26.1%). Compared with trials focusing on single subtypes (IBS-D vs. IBS-C; Figure 1H), probiotics were the most commonly investigated therapy for both. FMT was the second most studied therapy for IBS-D, while prebiotics were more frequently investigated for IBS-C.

Furthermore, we examined the publicly reported efficacy outcomes among completed trials (Supplementary Table 2). Efficacy results were available for 124 of the 305 trials (40.7%). Within this subset of trials with reported data, the following patterns were observed: (1) In trials reporting on probiotics (particularly multi-strain formulations and strains such as *Lactobacillus plantarum* 299v and *Bifidobacterium longum*), a high proportion (~86.5%) described symptom improvement in overall IBS severity, abdominal pain, bloating, or quality of life. (2) Fecal microbiota transplantation (FMT) showed significant efficacy in patients with refractory IBS-D (e.g., TrialTroveID-330317), which aligns with FMT being the second most frequently studied therapy for this subtype. (3) Marked heterogeneity was evident across studies—even when the same probiotic formulation was used, divergent conclusions could be reached in different trials (e.g., inconsistent outcomes regarding abdominal pain improvement with VSL#3 (see text footnote 1) in TrialTroveID-013151 versus TrialTroveID-100240).

## Discussion

The predominance of completed trials is consistent with the hypothesis that the clinical development of GMM therapies for IBS is relatively mature. The geographic concentration of trials in Asia could be explained, we hypothesize, by a combination of factors: the region’s large IBS population, its established probiotic industry, alongside potentially supportive policy environments and patient acceptance. For instance, it is plausible that substantial governmental support for functional food and probiotic preparation R&D, as seen in countries like China and India, could be one factor contributing to the accelerated growth of clinical trials in this field.

Shifting focus to the dominant therapeutic agents, the dual predominance of Phase II and Phase IV trials may reflects a bifurcated developmental trajectory: established therapies like probiotics are under post-marketing surveillance, while a growing pipeline of innovative interventions is undergoing initial efficacy validation. Within this landscape, probiotics maintain their status as the most mature and developed GMM approach. The leading position of *Lactobacillus* and *Bifidobacterium* is not only due to their extensive research background and recognized safety—exemplified by *Lactobacillus rhamnosus* GG and *Bifidobacterium animalis* subsp. *lactis* BB-12, which have been granted FDA GRAS status and are included on China’s list of probiotic strains for infant food^2^—but also to their functional mechanisms as evidenced in prior research. Beyond well-known metabolites like short-chain fatty acids (SCFAs), certain lactobacilli produce indole-3-lactic acid (ILA), which has been reported in the literature to activate the aryl hydrocarbon receptor (AhR) pathway to enhance intestinal barrier integrity and suppress inflammation (5). However, the efficacy of probiotics varies substantially across populations and studies. This heterogeneity is exemplified within our own dataset by the contrasting outcomes reported for the identical formulation, VSL#3 (e.g., TrialTroveID-013151 vs. TrialTroveID-100240), where one trial reported improvement in abdominal pain and the other did not. This observation aligns with a published meta-analysis of VSL#3 that showed no significant improvement in abdominal pain (6), although some pediatric studies have reported symptom relief. Such inconsistencies likely arise from multiple sources of clinical and methodological heterogeneity, including differences in primary endpoints (e.g., pain versus bloating), enrolled patient populations (e.g., general IBS versus specific subtypes or biomarker-defined subgroups), intervention protocols (dose, duration), and outcome measures. Another network meta-analysis also indicated substantial variations in the effects of different strains or formulations on various IBS outcomes—such as abdominal pain, bloating, and bowel movement frequency—further supporting the notion of efficacy heterogeneity (7). Therefore, future research should place greater emphasis on the interaction between host factors (e.g., genetics, diet, and microbial composition) and probiotic effects, moving away from a one-size-fits-all treatment strategy.

Beyond the predominant focus on probiotics, our landscape analysis also sheds light on the developmental status of other GMM approaches. Fecal microbiota transplantation (FMT) constituted the second-largest category of trials in our analysis. The majority of FMT trials were in Phase II, which may reflect that this field remains exploratory, potentially in the process of defining optimal administration protocols for IBS applications. For example, the transition from endoscopic delivery to oral capsules (as seen in Trial ID 565685 investigating a microbial capsule) could potentially improve patient adherence. This stands in contrast to the more established probiotic sector, which is characterized by a substantial number of Phase IV (post-marketing) studies.

Synbiotics, although representing a smaller proportion of trials, warrant attention. An observation from our dataset is that a considerable portion (47.1%) of synbiotic trials were Phase IV studies. This may indicate that certain specific synbiotic formulations have already reached the market or are undergoing post-marketing surveillance to verify their long-term safety and efficacy in real-world settings.

Similarly, the number of trials investigating postbiotics was limited. However, emerging evidence beyond the scope of our analysis suggests their promise. For instance, a randomized controlled trial in preterm infants demonstrated that a heat-inactivated *Bifidobacterium* postbiotic modulated the gut microbiota to a comparable extent as its live probiotic counterpart, while being able to circumvent risks associated with live bacteria (8). This suggests that postbiotics could potentially represent a safer alternative therapeutic strategy.

Regarding prebiotics, our analysis noted that research in this area is sometimes intertwined with dietary interventions. For example, Trial ID 376087 combined a prebiotic with a low-FODMAP diet. While such a design aligns with real-world clinical practice, it complicates the interpretation of results by making it difficult to isolate the specific contribution of the prebiotic component. Future trial designs could benefit from more precise stratification or stricter control of dietary backgrounds to clarify the independent efficacy of prebiotics in treating IBS.

This heterogeneity in therapeutic approaches across different GMM categories further underscores the need for a personalized strategy, a principle that is also reflected in our analysis of IBS subtypes. The observed divergence in research focus across IBS subtypes aligns with their distinct pathological frameworks. Specifically, the more frequent investigation of FMT in IBS-D trials is consistent with the subtype’s association with gut microbiota dysbiosis (9), for which FMT represents a direct strategy to test ecological remodeling and barrier restoration (10). Conversely, the greater research emphasis on prebiotics in IBS-C coincides with the exploration of interventions designed to modulate microbial metabolism, including the promotion of SCFA production, which may influence gut motility (11). It is critical to emphasize that these associations reflect current investigative priorities and trends in clinical trial design, not established therapeutic superiority, given that our study assesses trial frequency rather than comparative efficacy.

Looking forward, breakthroughs in basic research are accelerating their translation into clinical practice. For example, the emerging strain *Akkermansia muciniphila* (a mucin-degrading bacterium) can enhance intestinal barrier function and exert anti-inflammatory effects, and its clinical trial for the treatment of IBS has entered Phase III (TrialTroveID-511100). This translational bridge between cutting-edge exploration and clinical practice is expected to expand the repertoire of therapeutic options for IBS treatment.

This study has several limitations. First, although the Trialtrove database integrates data from multiple primary registries, including ClinicalTrials.gov and the WHO International Clinical Trials Registry Platform (ICTRP), the use of a single database may still have led to the omission of some relevant trials. Examples include certain exploratory early-phase studies, trials registered exclusively on regional platforms, or those sponsored by non-commercial entities. This could affect the comprehensiveness of the present landscape analysis. A further, and crucial, limitation is that the interpretability of efficacy outcomes is constrained by the incompleteness of registry-reported data. We found that efficacy results were publicly available for only 40.7% (124/305) of the included trials. Consequently, the summary of efficacy—such as the approximately 86.5% symptom improvement rate reported for probiotics—is based on an incomplete subset of all completed trials. Moreover, these reported data are heterogeneous and not centrally adjudicated. Therefore, any interpretation regarding efficacy should be regarded as preliminary, as it does not account for trial quality, risk of bias, or effect size.

In summary, probiotic therapy remains the dominant approach in the current field, while FMT also has advantages, especially in the more concerning IBS-D subtype. Future research should focus on developing personalized microbiota-based therapies tailored to individual patient characteristics, as well as integrating multiomics approaches (e.g., combining metagenomic and metabolomic biomarkers), to guide precise microbial formulation selection. Emerging therapies such as EBX-102-02—a novel full-spectrum microbiome therapeutic currently in phase II trials (TrialTroveID-565759)—represent promising new directions. Integrating these innovative strategies with personalized approaches will be essential for the continued evolution and improvement of IBS care, moving incrementally toward the goal of precision medicine.

## Footnotes

1 It should be noted that post-2016 VSL#3 probiotic formulation differs from the De Simone formulation, which was commercially available under the trademark VSL#3 only until 2016 (12).

2 It is critical to distinguish that these designations confirm safety in specific food contexts, not therapeutic efficacy or approval for treating IBS.
